# Supplementary material for: Eradication of Saccharomyces cerevisiae by Pulsed Electric Field Treatments
Source: Microorganisms. 2020 Oct 29;8(11):1684. doi: 10.3390/microorganisms8111684 (PMC7692574; doi:10.3390/microorganisms8111684)
Supplement: Supplementary file 1 [file microorganisms-08-01684-s001.pdf]

Suplumentary material S1

Photos of electroporator chamber and the instrument:

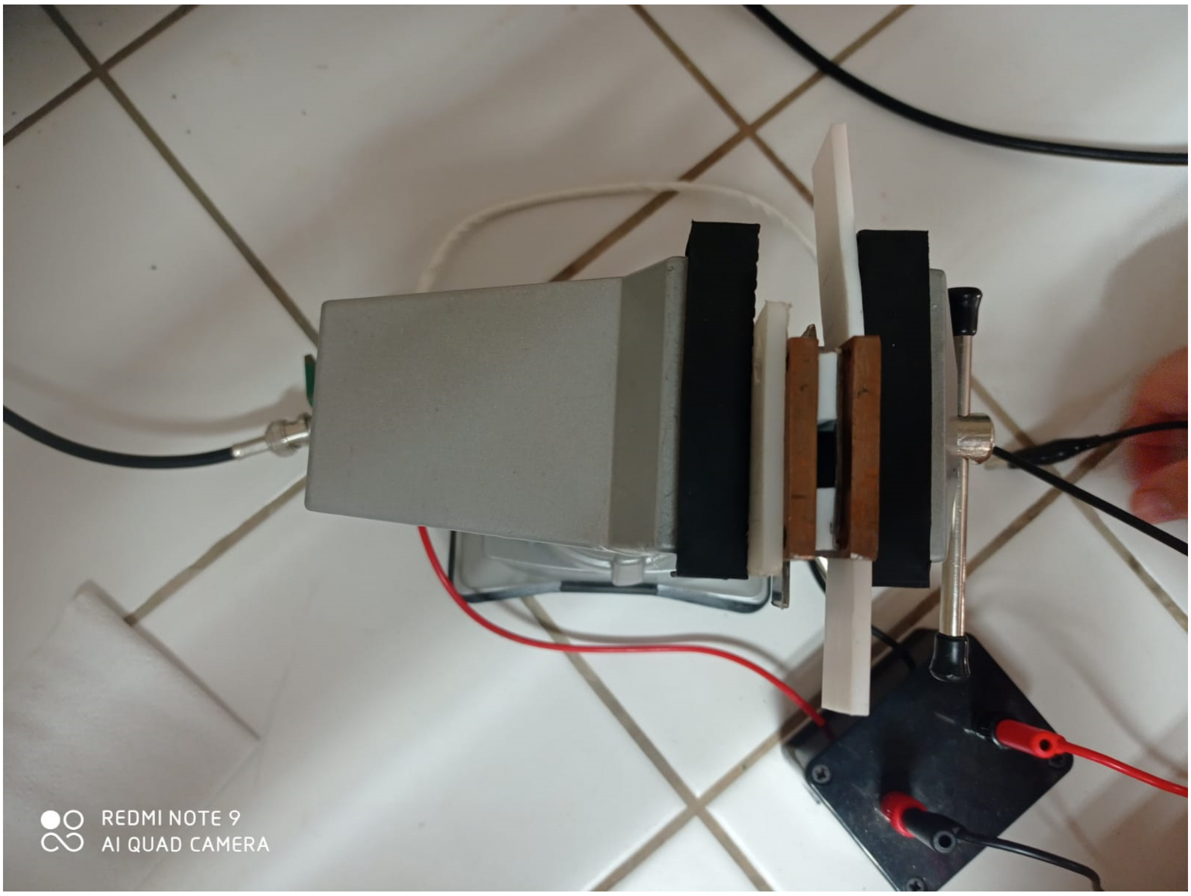

Figure 1The electroorator chamber above view.

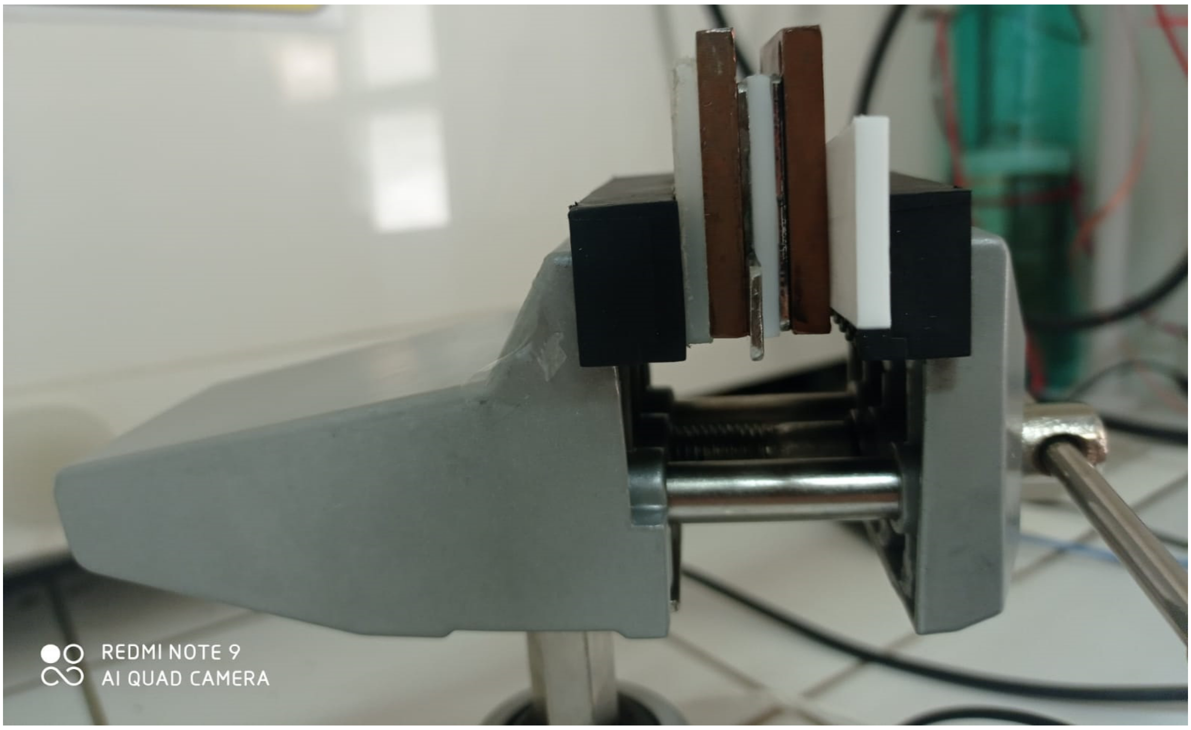

Figure 2 The electroorator chamber side view.

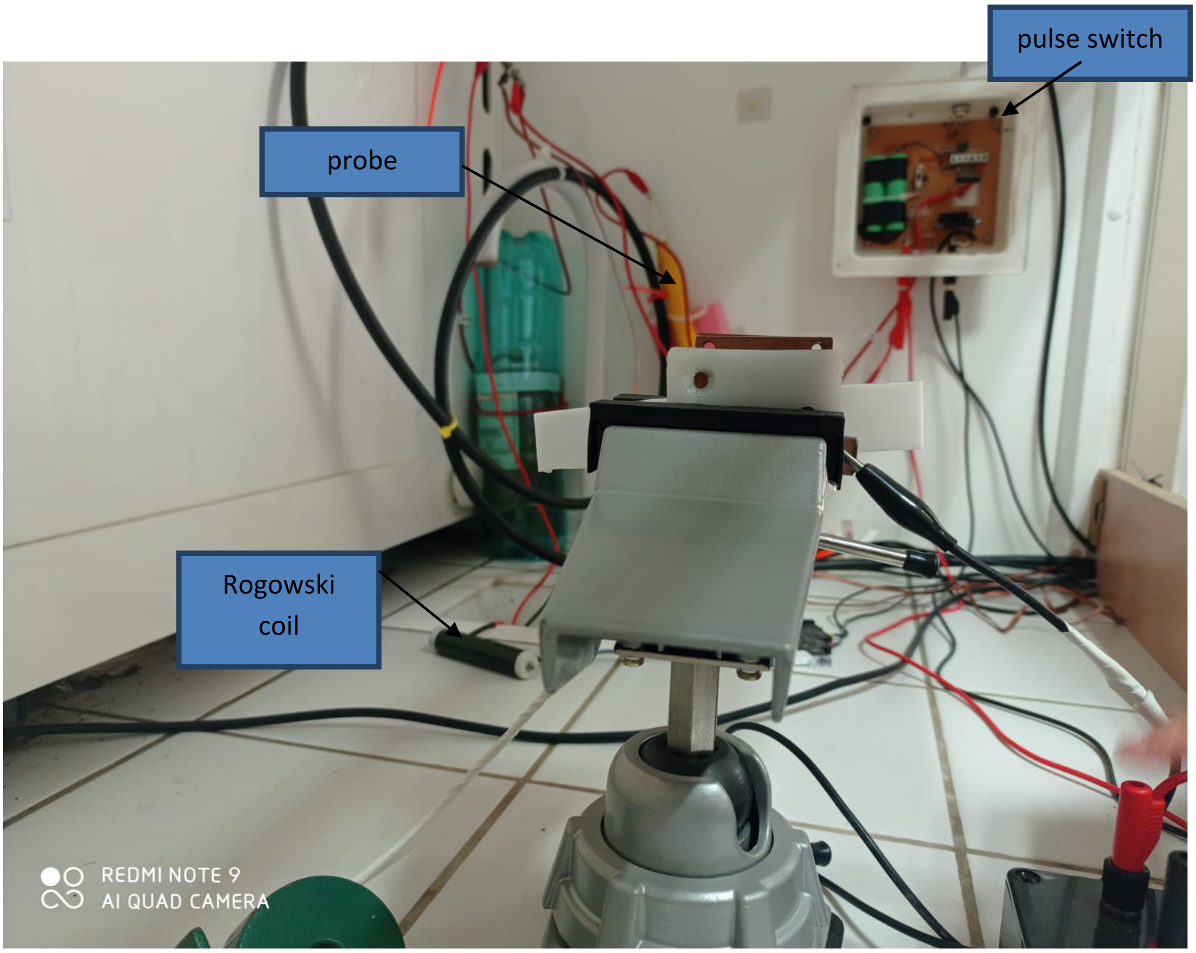

Figure 3 The instrument

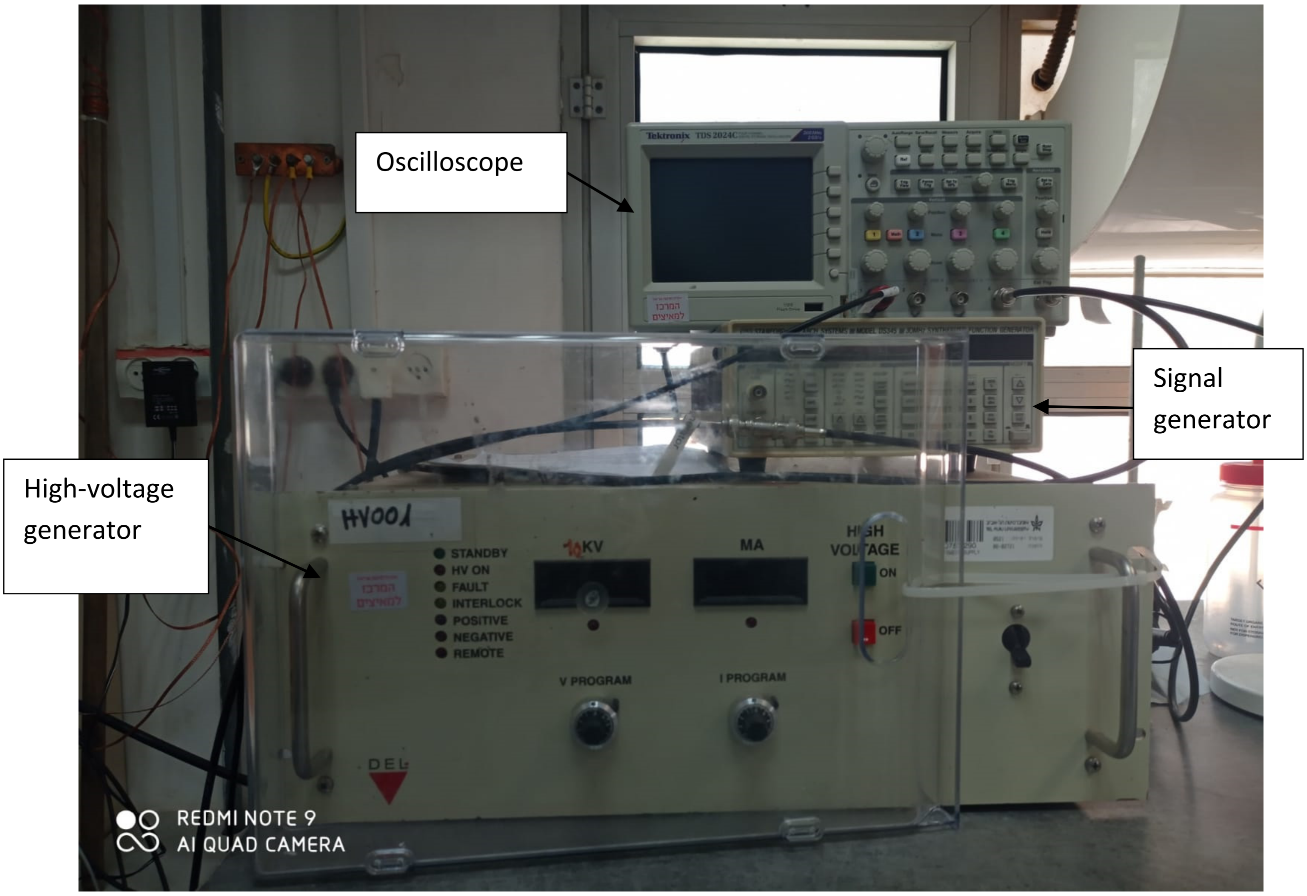

Figure 4The instrument

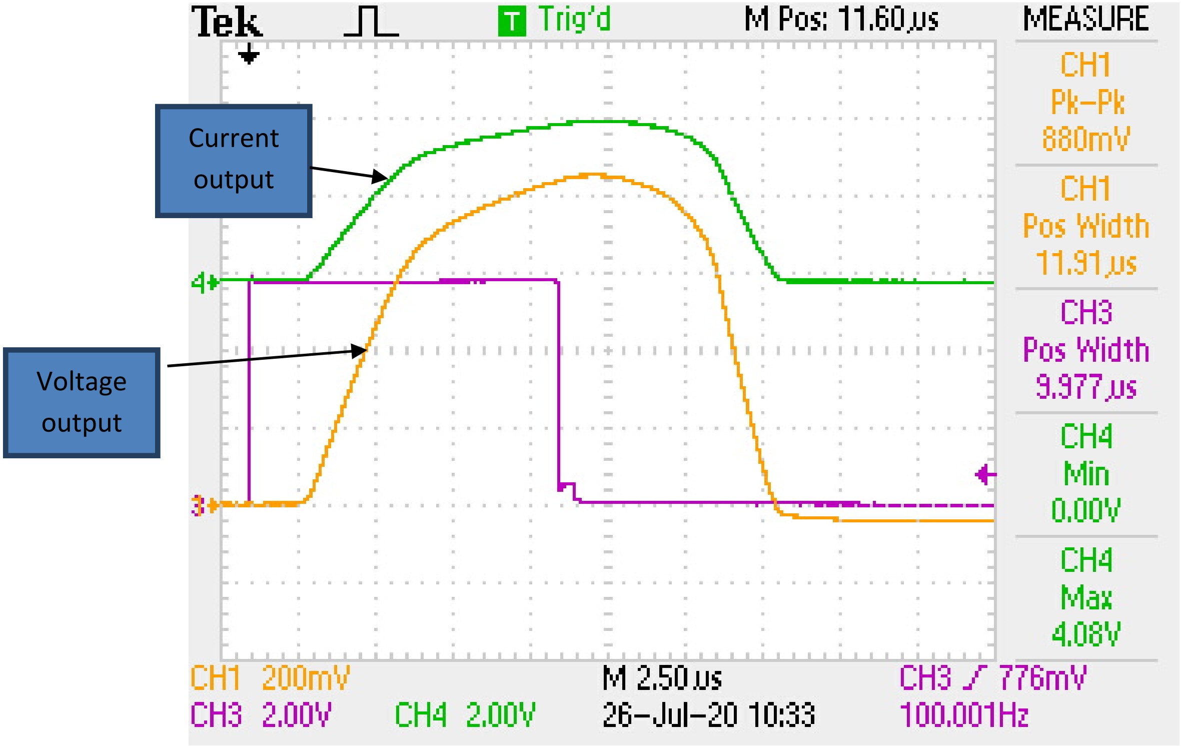

Figure 5 The shape (voltage and current) of pulse on the cuvette filled with PBS (the highest conductivity).

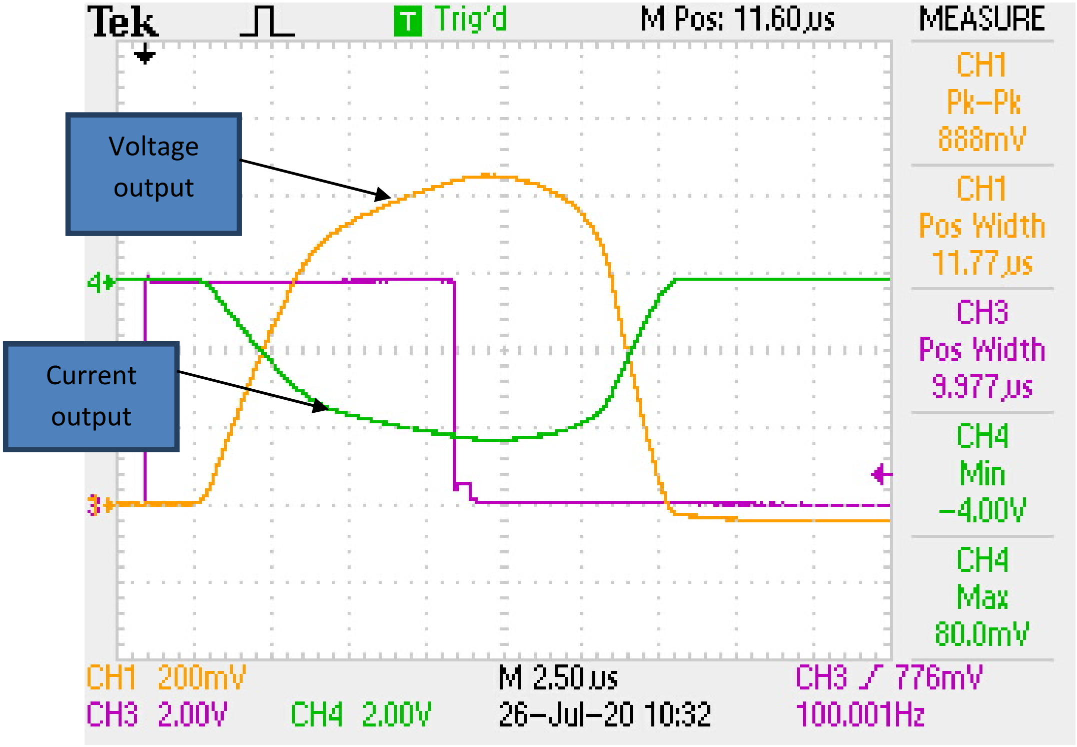

Figure 6 The shape (voltage and current) of pulse on the cuvette filled with PBS (the highest conductivity). negative polarity.
